# Fatigue Performance Analysis of Weathering Steel Bridge Decks Under Residual Stress Conditions

**DOI:** 10.3390/ma18173943

**Published:** 2025-08-22

**Authors:** Wenye Tian, Ran Li, Tao Lan, Ruixiang Gao, Maobei Li, Qinyuan Liu

**Affiliations:** 1 School of Civil Engineering, Xian University of Architecture & Technology, Xi’an 710055, China; 13253132913@163.com (W.T.); lmb3021@163.com (M.L.); l547790690@163.com (Q.L.); 2 CSIC International Engineering Co., Ltd., Beijing 100121, China

**Keywords:** residual stress, fatigue crack propagation, finite element simulation, vehicular load effects, welded joints

## Abstract

The growing use of weathering steel in bridge engineering has highlighted the increasing impact of fatigue damage caused by the combined effects of welding residual stress and vehicular loading. This study investigates the fatigue performance of Q500qENH weathering steel bridge decks by proposing a coupled analysis method for residual stress and fatigue crack growth, utilizing collaborative simulations with Abaqus 2023 and Franc3D 7.0. An interaction model integrating welding-induced residual stress fields and dynamic vehicular loads is developed to systematically examine crack propagation patterns in critical regions, including the weld toes of the top plate and the weld seams of the U-ribs. The results indicate that the crack propagation rate at the top plate weld toe exhibits the most rapid progression, reaching the critical dimension (two-thirds of plate thickness) at 6.98 million cycles, establishing this location as the most vulnerable failure point. Residual stresses significantly amplify the stress amplitude under tension–compression cyclic loading, with life degradation effects showing 48.9% greater severity compared to pure tensile stress conditions. Furthermore, parametric analysis demonstrates that increasing the top plate thickness to 16 mm effectively retards crack propagation, while wheel load pressures exceeding 1.0 MPa induce nonlinear acceleration of life deterioration. Based on these findings, engineering countermeasures including welding defect control, optimized top plate thickness (≥16 mm), and wheel load pressure limitation (≤1.0 MPa) are proposed, providing theoretical support for fatigue-resistant design and maintenance of weathering steel bridge decks.

## 1. Introduction

Q500qENH high-strength weathering steel forms a dense oxide rust layer through the Cu-Cr-Ni alloy system, combining high strength with exceptional weather resistance [1]. Its integration with orthotropic steel bridge decks, leveraging advantages such as light weight, high strength, and modular construction, has made it a preferred solution for high-performance bridges in plateau environments [2]. However, the coupling effect of welding residual stress and cyclic vehicular loads generates multiaxis asymmetric stress fields at critical welded joints, such as the top plate-to-U-rib weld toes [3]. Studies indicate that residual tensile stresses induced by welding thermo-elastoplastic deformation can exceed 80% of the material yield strength, with significant stress concentration at weld toes [4]. Dynamic loads induce stress redistribution and relaxation effects through alternating stress coupling [5], leading to deviations of 20–40% in fatigue life assessments using traditional nominal stress methods [6].

Under the framework of fracture mechanics, Griffith’s energy release rate criterion established the critical conditions for unstable crack propagation [7]. Building on Irwin’s stress intensity factor theory [8], researchers have found that welding residual stresses significantly influence fatigue life by altering the crack-tip stress field. Lee’s thermomechanical coupled simulations demonstrated that residual tensile stresses amplify equivalent alternating stress amplitudes by over 20% [9]. XFEM analyses revealed that residual stress gradients modify crack closure effects [10], while Backer’s elastic fracture mechanics model showed stress amplitude fluctuations increasing by 20–40% [11]. Regarding welding process impacts, Cui Chuang et al. developed an OSD dynamic evolution model to characterize the residual stress gradient in top plate-to-U-rib welds [12]. Zhong Wen proposed a stress relaxation coefficient model, indicating an initial stress relaxation rate of up to 50% during cyclic loading [13], while optimized welding parameters reduced residual stress peaks by over 30% [14].

Nevertheless, existing studies predominantly focus on conventional steels, leaving critical gaps in understanding the fatigue damage mechanisms of high-strength weathering steels like Q500qENH under complex stress-coupled fields:

(1) The high yield strength (556 MPa at ambient temperature) necessitates systematic investigations into the modification laws of welding residual stress fields on crack driving forces (e.g., stress intensity factors), as current models exhibit significant evaluation deviations;
(2) The sensitivity of fatigue life to initial crack dimensions (size, angle, etc.) under residual stress–dynamic load coupling remains unquantified, hindering the refinement of fatigue-resistant design specifications.

**Figure 1:**
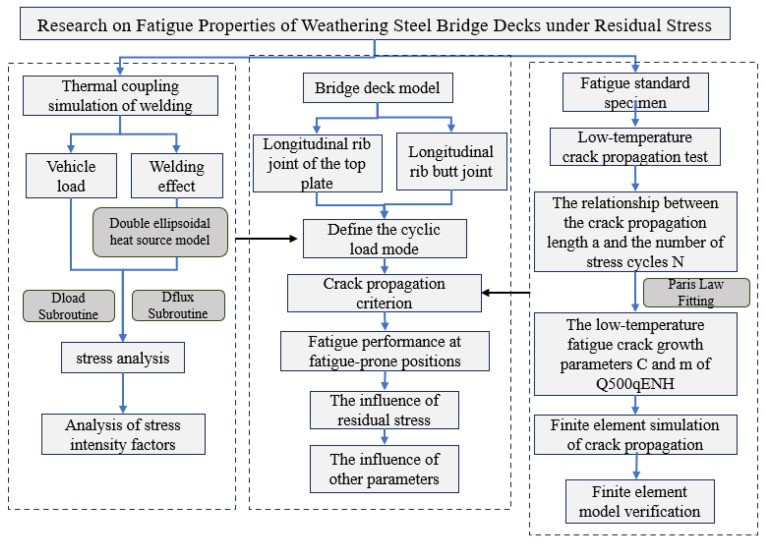
The technical flow of this study.

To address these issues, this study proposes a thermo-elastoplastic finite element simulation–multiaxial fatigue damage model–elastoplastic fracture mechanics integrated methodology. A dynamic coupling model of residual stress and vehicular stress is established to analyze the evolution of stress intensity factors in critical regions (e.g., top plate weld toes, U-rib weld seams). The nonlinear relaxation of residual stress under tension–compression cycling and its degradation mechanism on crack propagation thresholds are elucidated. Parameter control indices based on crack driving forces and wheel load pressures are subsequently formulated. The technical flow of this study is illustrated in Figure 1.

## 2. Establishment of the Model for Bridge Deck Welding and Fatigue Crack Propagation

### 2.1. Establishment of the Model for Bridge Deck Welding

#### 2.1.1. Definition of Thermodynamic Parameters of Q500qENH Weathering Steel

In order to obtain the thermodynamic parameters of Q500qENH weathering steel, this paper uses the analysis software JMatPro 7.0 based on the chemical composition of the steel to simulate and calculate the changes in the thermal parameters of the steel during the temperature variation process. The chemical composition of Q500qENH weathering steel is shown in Table 1.

**Figure 2:**
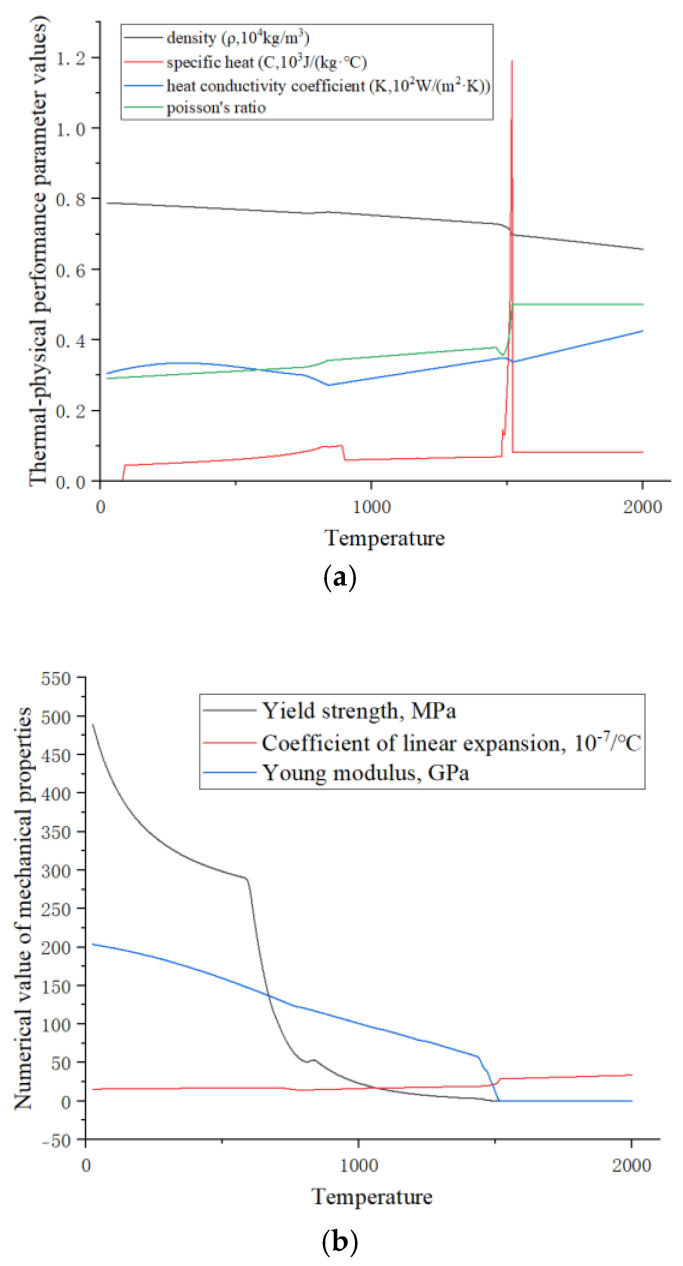
(**a**) Thermal–physical properties of Q500qENH steel and (**b**) thermodynamic properties of Q500qENH steel.

The chemical composition of Q500qENH weathering steel was input into the analysis software JMatPro, and the relationship between the thermodynamic parameters of Q500qENH weathering steel and temperature was analyzed, as shown in Figure 2.

#### 2.1.2. Layout of Welding Heat Sources

The arrangement of the heat source for the bridge deck welding selected in this paper is a double-ellipsoidal heat source model. The double-ellipsoidal heat source model can better simulate the state of the welding molten pool. The welding process mainly adopts CO_2_-gas-shielded welding.

By calling the Dflux subroutine template in the Abaqus help documentation and based on the welding heat source parameters in Table 2, the Dflux subroutine required for this model was compiled using the Fortran language, and the subroutine was substituted into the finite element welding model of the weathering steel bridge deck to simulate the welding process of the weathering steel bridge deck.

#### 2.1.3. Establishment of the Welding Model for Weathering Steel Bridge Decks

**Figure 3:**
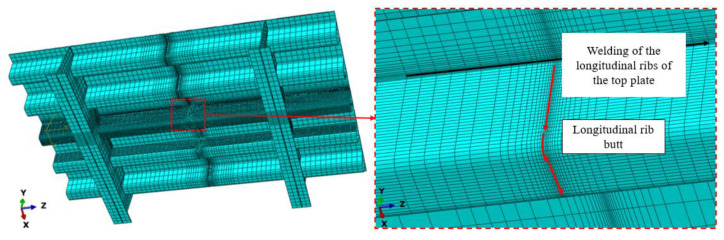
Grid division of the welding model for weathering steel bridge decks.

Welding simulations were conducted for the longitudinal rib welding of the top plate and the longitudinal rib butt welding. The length of the longitudinal rib weld of the top plate was 800 mm. Two temperature–displacement coupling analysis steps were set for the longitudinal rib welding of the top plate. Step 1 was the welding step, and step 2 was the cooling step. Four temperature–displacement coupling analysis steps were set for the longitudinal rib butt joint. Analysis steps 1, 2, and 3 were the welding steps for the longitudinal rib web, the longitudinal rib flange, and the junction, respectively, and analysis step 4 was the cooling step. The welding speed was set to 10 mm/s, the cooling step time was set to 3000 s, the unit type was a C3D8RT solid unit, the initial temperature was set to 20 °C, the convective heat transfer coefficient was 10 W/(m^2^·K), the radiation factor, or emissivity, was 0.8, the Stefan–Boltzmann constant is 5.67 × 10^−8^ W/(m^2^·K^4^), and absolute zero is −273.15 °C. The grid division of the weathering steel bridge deck welding model is shown in Figure 3. Fixed constraints are applied to the four sides of the finite element model of the weathering steel bridge deck as boundary conditions.

**Figure 4:**
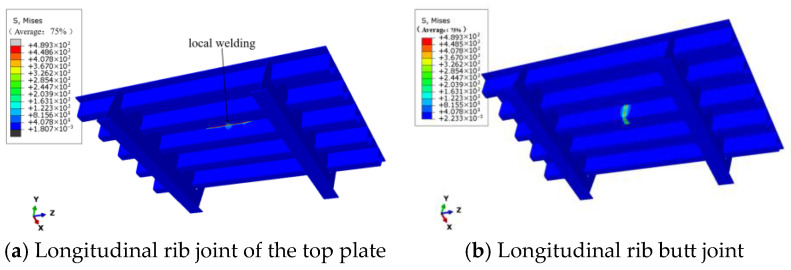
Residual stress distribution at welded joints of weathering steel bridge decks.

As can be seen from Figure 4, the residual stress is mainly distributed along the weld seam. The center of the weld seam is the molten zone, where the residual stress is the greatest. The residual stress near the welding center even reaches the yield strength of Q500qENH weathering steel.

**Figure 5:**
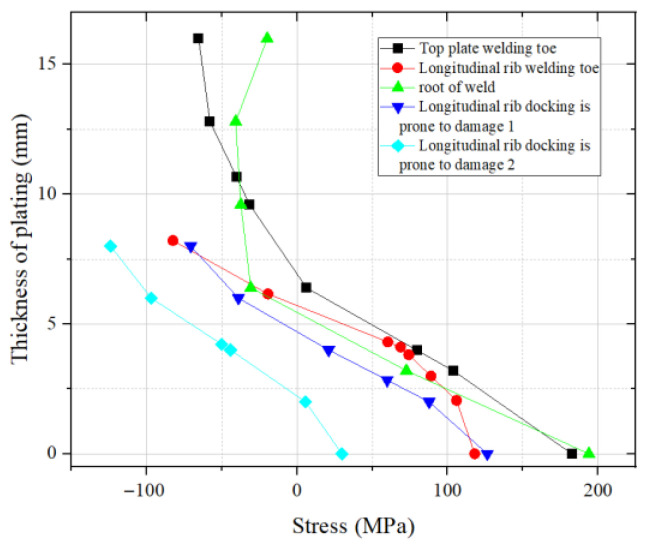
Distribution of residual stress at vulnerable positions of welded joints.

For the fatigue crack propagation of bridge decks, the residual stress components at the welded nodes perpendicular to the crack surface direction should be given particular attention. Figure 5 shows the distribution of residual stress in the thickness direction at the vulnerable positions of the welded joints of weathering steel bridge decks.

As can be seen from Figure 5, the distribution of residual stress along the thickness direction of the plate is mainly tensile stress, and the residual tensile stress is mainly concentrated on the lower side of the plate, while the residual compressive stress is mainly concentrated on the upper side.

### 2.2. Fatigue Crack Propagation Model Establishment for Bridge Deck

**Figure 6:**
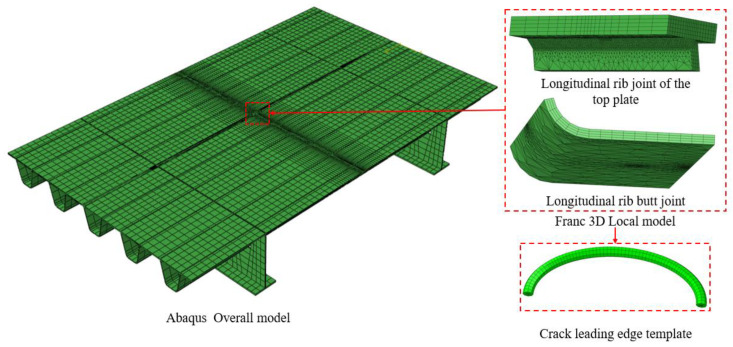
Finite element model of weathering steel bridge deck.

Based on collaborative simulation using Abaqus 2023 and Franc3D 7.0, a fatigue crack propagation model coupled with vehicular loads and welding residual stresses was established. First, the global model of the bridge deck was developed in Abaqus (see Figure 6).

The welding residual stress field was obtained through thermo-elastoplastic numerical simulation, with welding parameters strictly set in accordance with the heat input control requirements specified in the AWS D1.5 Bridge Welding Code [14]. Upon completing the thermo-elastoplastic analysis of the welding process and static vehicular load analysis, the residual stress field was extracted and imported into the crack propagation zone in Franc3D [15]. An initial semi-elliptical crack was defined via adaptive meshing, with parameters set based on welding defect statistical results [16]: minor axis depth = 0.3 mm, major axis length = 0.5 mm, and depth-to-width ratio = 0.3.

**Figure 7:**
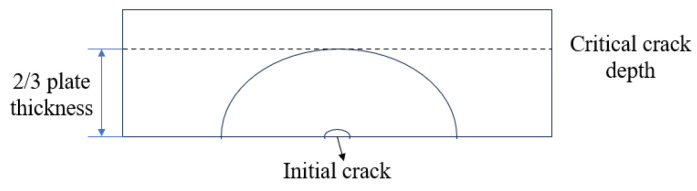
Semi-elliptical surface crack.

Subsequently, the stress intensity factor (SIF) under the superposition of residual stress and vehicular loads was calculated using the M-integral method. As the cyclic stress iterations increased, the fatigue crack gradually propagated until reaching the critical crack dimension. Beyond this dimension, crack propagation accelerated significantly, with crack depth rapidly approaching the plate thickness [17]. Therefore, structural failure is generally considered to occur when the crack attains the critical dimension. For steel bridge decks, the critical crack dimension typically ranges from 1/2 to 2/3 of the plate thickness. As shown in Figure 7, this study selected 2/3 of the plate thickness as the critical crack dimension.

## 3. Finite Element Validation Analysis

### 3.1. Finite Element Verification Analysis of Welding

#### 3.1.1. Geometric Model of the Welded Part and Material Property Parameters

**Figure 8:**
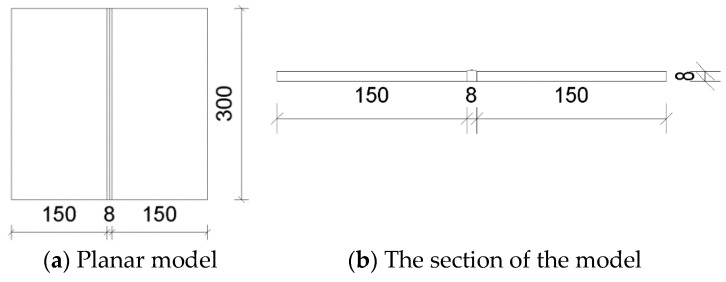
Specimen dimensions.

The size of the welded steel plate used in the verification experiment is selected as 300 mm × 150 mm. Two welded parts are spliced and welded, and the bevel is an I-shaped bevel with a width of 8 mm. The geometric dimensions of the steel plate components are shown in Figure 8.

In the finite element numerical simulation of welding, due to the huge range of temperature changes, the influence of temperature on the mechanical properties of materials is also significant. The steel grade used in this chapter is Q235B, which belongs to low-alloy structural steel. The physical performance parameters of the steel are shown in Table 3.

#### 3.1.2. Finite Element Mesh and Thermal Load Application

When selecting the element type for the temperature field analysis model, the principle of regular division with a heat transfer function is adopted. Considering the characteristics of uneven heating and large temperature gradient changes during the welding process, the eight-node hexahedral solid element DC3D8 is chosen.

The grid division selected the eight-node hexahedral thermal element DC3D8. The weld and heat-affected zone were densified to a minimum size of 1 mm, and the grid is divided through transition. The length and width of the grid far from the weld seam are 12 mm, and the thickness is 4 mm A total of 33,400 elements were divided.

In the model of steel plate butt welding, the initial temperature of the weldment and the environment is set at a room temperature of 20 °C, the convective heat transfer coefficient is 10 W/(m^2^·°C), and the radiation factor, that is, the emissivity, is 0.8. And absolute zero is set at −273.15 °C, with a radiation coefficient of 5.67 × 10^−8^ W/(m ^2^·°C^4^).

#### 3.1.3. Boundary Conditions

**Figure 9:**
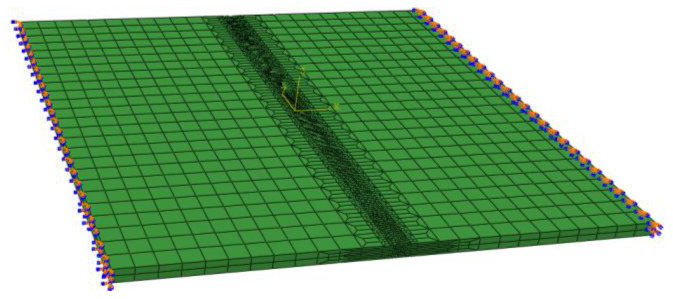
Constraint condition diagram of the finite element model.

To prevent deformation and movement of the welded parts during the welding process due to “thermal expansion and contraction”, fixed constraints are applied to the six degrees of freedom including translational and rotational movements in the X, Y, and Z directions on the left and right cross-sections of the welded part model. In the fixture release analysis step, all the constraints on the right side are released. The constraint conditions are shown in Figure 9.

#### 3.1.4. Welding Residual Stress Experiment and Finite Element Comparison

**Figure 10:**
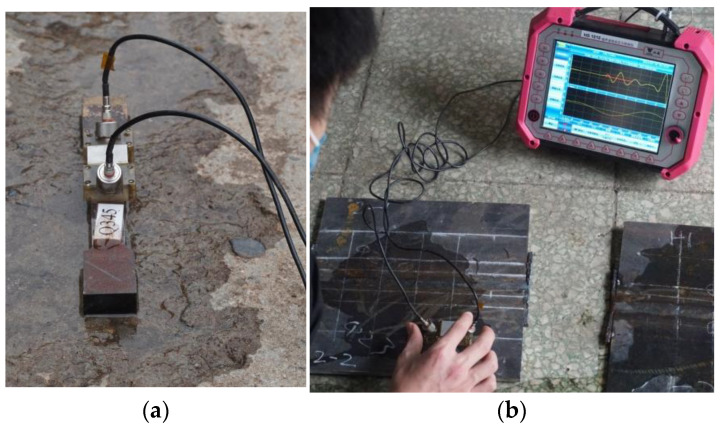
Ultrasonic residual stress detection. (**a**) Ultrasonic propagation of zero-stress test block. (**b**) Ultrasonic propagation of the welded part.

The welding residual stress of an 8 mm thick welded piece made of Q235B material was compared between the test and the finite element numerical simulation. The residual stress testing experiment employs ultrasonic detection technology. By setting up measurement points along three pre-determined paths and combining the measurement of calibrated specimens, the distribution of welding residual stress is obtained. The specific process is as follows: Firstly, tensile load is applied to the calibrated specimen (zero-stress test block), and the internal stress value and the corresponding ultrasonic propagation time are recorded in real time. Subsequently, the surface of the welded part to be tested is ground to reduce the error. Finally, using the HS 1010 ultrasonic residual stress detector [Manufacturer: Wuhan Zhongke Innovation Technology Co., Ltd.; Sourced from: Wuhan, China], point-by-point measurements are carried out according to the positions of the measurement points along the preset path (see Figure 10).

**Figure 11:**
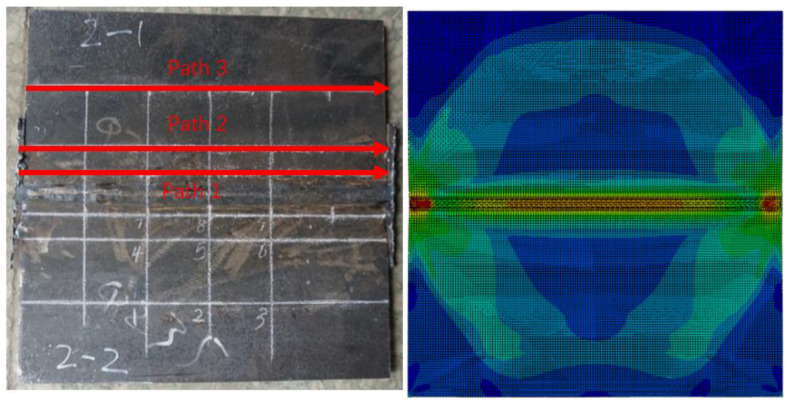
Measurement of path 1–3 and the finite element residual stress field.

In order to analyze the differences in and distribution of residual stress in 8 mm thick welded components, the measurement paths 1–3 of the welding test specimens and the residual stress field simulated by the finite element method are shown below, as depicted in Figure 11.

**Figure 12:**
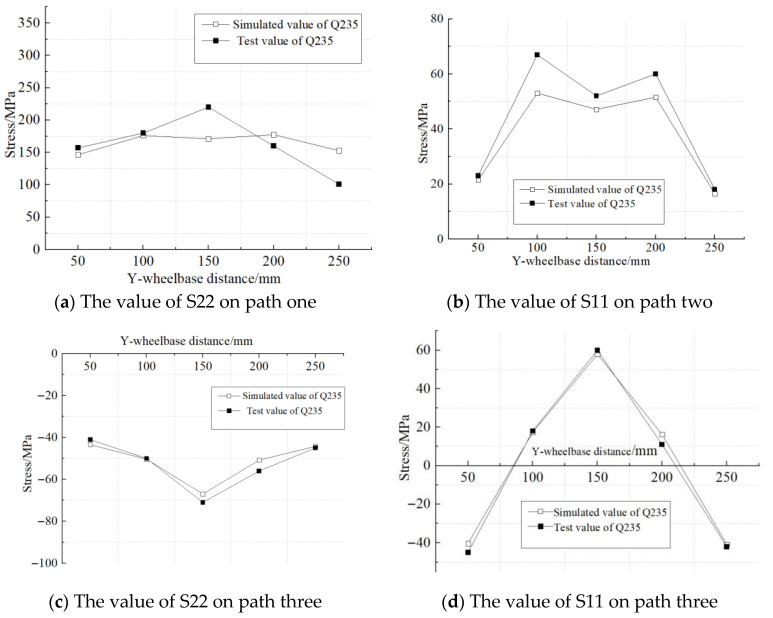
Residual stress along the weld direction.

It can be easily seen from the above figure that the residual stress of each welded part is mainly distributed in the weld seam and the areas at both ends of the weld seam. The longitudinal S22 and transverse S11 residual stresses distributed along the Y-axis in the welding test specimens were taken and compared with the residual stress values obtained from the finite element numerical simulation, as shown in Figure 12.

As can be seen from Figure 12, the variation trend of the welding residual stress measured by the finite element simulation and the test in the weld direction is basically consistent. Among them, there are certain random fluctuations in the test values, mainly due to the influence of the surface roughness of the welded part on the measurement accuracy. On path three, far from the weld seam, the longitudinal stress (S11) and transverse stress (S22) of the Q235B weldment are both below 70 MPa. However, on path one, adjacent to the weld seam, the stress value of S22 significantly increased, indicating that the welding residual stress was concentrated in the weld seam and its adjacent areas. Overall, the consistency between the simulation results and the test data verifies the reliability of the finite element model. This modeling method can be further applied to the prediction research of welding residual stress of the Q500qENH bridge deck.

### 3.2. Finite Element Verification and Analysis of Fatigue Model

The finite element validation analysis was conducted based on fatigue crack propagation tests of Q500qENH weathering steel compact tension (CT) specimens, designed according to the GB/T 6398-2017 [18] standard (specimen width W = 50 mm, initial crack length a_0_ = 10 mm). Loading was applied at −40 °C and 20 °C (stress ratio R = 0.1, load amplitude ΔP = 11 kN). Finite element models were developed to calculate the crack propagation life, and the results were compared with experimental data to validate the methodology.

**Figure 13:**
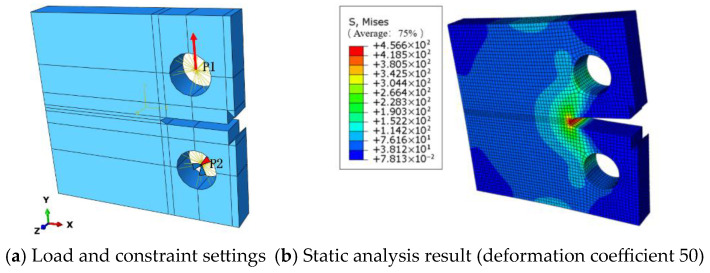
CT static analysis of the finite element model of CT specimens.

CT Specimen Crack Propagation Model: Based on elastic theory, a collaborative simulation using Abaqus and Franc3D was employed for finite element analysis of CT specimen fatigue crack propagation. The accuracy of the finite element model was verified by comparing numerical results with experimental data. First, a parametric CT specimen model was created in Abaqus. Two reference points (P1 and P2 shown in Figure 11) were defined on the inner surface of the bolt hole with kinematic coupling constraints established. A fixed constraint was applied to P2, while a concentrated axial load of 12.2 kN was applied to P1 (loading configuration shown in Figure 13). Static analysis was performed to obtain structural responses. The numerical results revealed a maximum stress concentration of 456.6 MPa at the notch tip, which remained below the material yield limit, confirming the applicability of linear elastic analysis. This model successfully predicted stress distribution characteristics and crack initiation locations, providing reliable initial stress field data for subsequent Franc3D crack propagation simulations.

**Figure 14:**
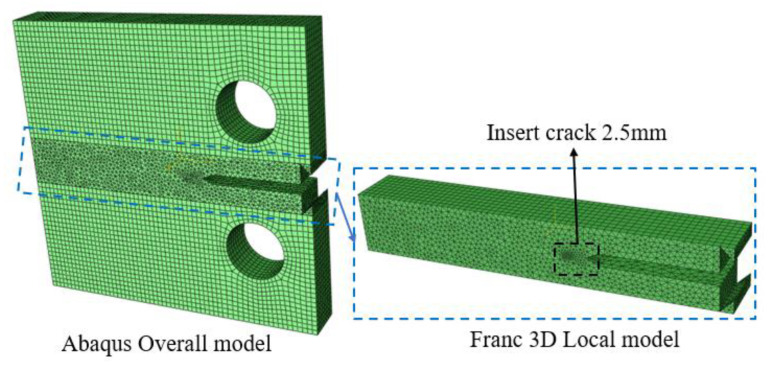
Adaptive meshing of CT specimens.

The static analysis-derived model was imported into Franc3D, and a 2.5 mm pre-existing crack was inserted into the local model region. Subsequently, meshing was performed, as illustrated in Figure 14. The 12.2 kN load from the static analysis was designated as the maximum cyclic load, with a stress ratio R = 0.1, resulting in a load amplitude of 11.0 kN, consistent with the experimental conditions. The crack propagation criterion employed the Paris law, incorporating experimentally derived crack propagation parameters *C* and *m*.

At 20 °C, the Paris crack propagation criterion is expressed as

(1) $$da/dN=9.268\times 10^{-11}{\left(\Delta K\right)}^{2.158}$$

At −40 °C, the Paris crack propagation criterion is defined as

(2) $$da/dN=6.966\times 10^{-12}\left(\Delta K\right)$$

**Figure 15:**
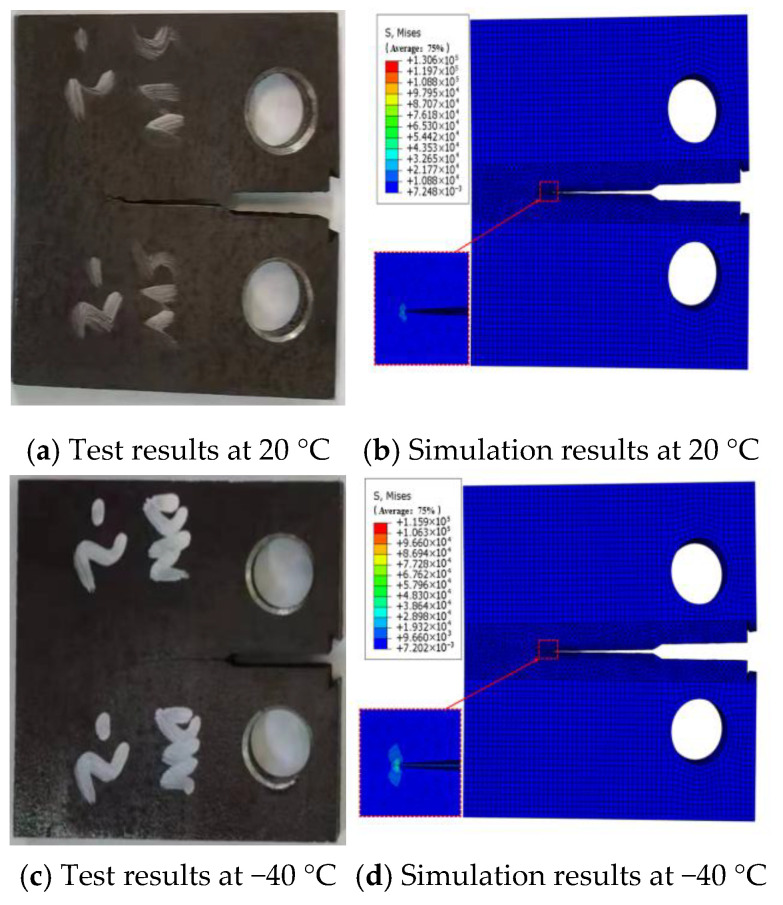
CT simulation comparison of CT sample tests.

After inserting the pre-existing crack in Franc3D, meshing of the local model region was completed, and cyclic loading patterns and crack propagation criteria were configured. Subsequently, the local model was merged with the remaining structure and imported into Abaqus for computation. Figure 15 illustrates a comparative analysis of the fracture morphology between the experimental and finite element simulation results for CT specimen fatigue crack propagation rates at 20 °C and −40 °C. Under uniaxial cyclic loading with a stress ratio R = 0.1, both the experimental and numerical simulations exhibited nearly linear crack propagation characteristics. Experimental observations identified minor plastic deformation zones at the crack tip, while the finite element model distinctly captured high-stress-concentration features at the crack tip through stress contour plots.

**Figure 16:**
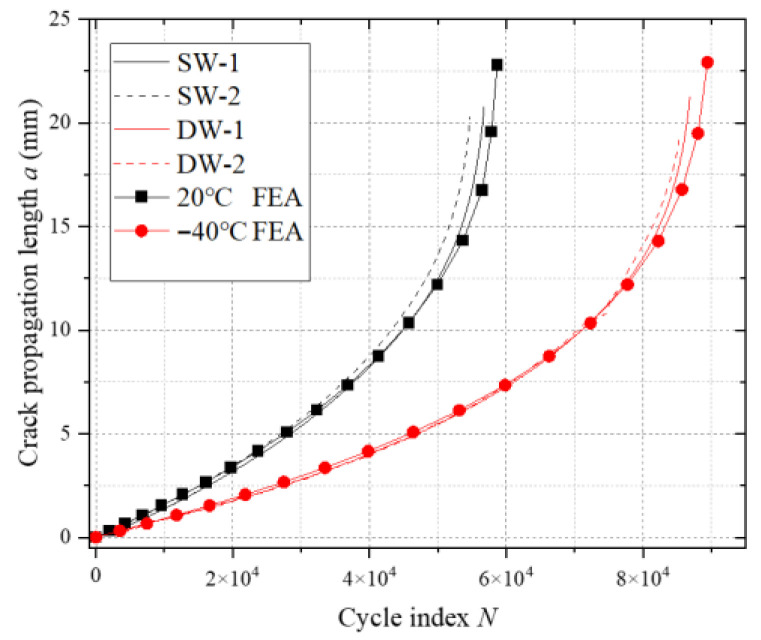
Comparison of CT sample tests and simulated a-N curves.

The comparative results demonstrated high consistency between the simulated and experimental crack propagation paths and final fracture morphologies under both temperature conditions, particularly with errors of less than 8% in crack length–cycle number curves (see Figure 16). This cross-scale alignment of experimental data and numerical outcomes confirms the high accuracy of the finite element analysis methodology established in this study.

## 4. Fatigue Analysis Under Coupled Residual Stress Effects

**Figure 17:**
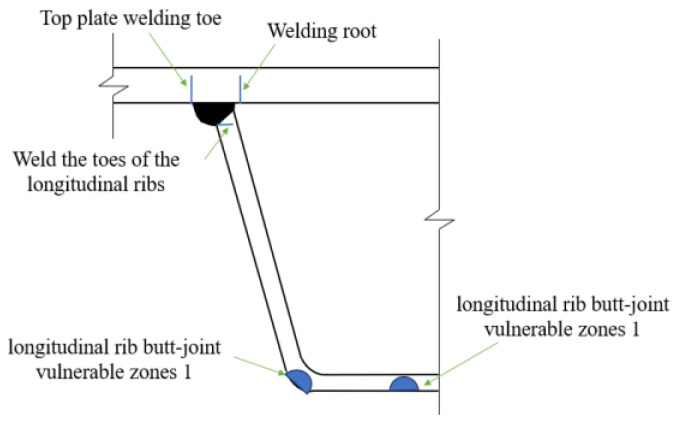
Vulnerable positions of welded joints on weathering steel bridge decks.

With reference to study [19] on stress analysis conducted on welded joints of weathering steel bridge decks, the critical vulnerable regions—including the top plate weld toe, weld root, longitudinal rib weld toe, and longitudinal rib butt-joint vulnerable zones 1 and 2 (see Figure 17)—were identified. Building on the coupling effects of vehicular loads and residual stresses, this section conducts a fatigue crack propagation analysis for the five vulnerable positions on the weathering steel bridge deck.

### 4.1. Initial Crack Size

The initial crack size significantly influences the stress intensity factor at the crack front, thereby affecting the fatigue crack propagation process. For bridge deck welded joints, initial cracks are typically induced by welding defects, with their dimensions and angles exhibiting inherent randomness due to variations in welding processes and operational conditions. This section investigates the impact of different initial crack sizes on fatigue crack propagation. Table 4 lists the initial crack configurations with varying depth-to-width ratios. Crack types C1, C3, and C5 adjust the depth-to-width ratio by modifying the initial crack width (major axis), while C2, C3, and C4 achieve this by altering the initial crack depth (minor axis).

**Figure 18:**
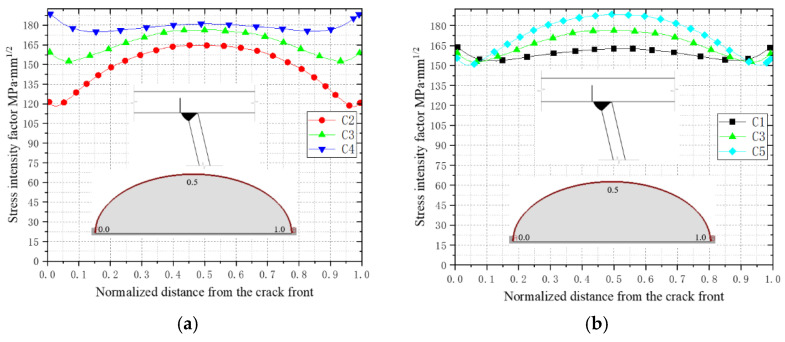
Stress intensity factors for initial cracks of different sizes. (**a**) The changes in stress intensity factors of C2, C3, and C4. (**b**) The changes in stress intensity factors of C1, C3, and C5.

Figure 18 illustrates the influence of different initial crack sizes (C1–C5) at the top plate weld toe on the stress intensity factor (SIF) at the crack front. Comparing C2, C3, and C4 (with progressively increasing initial crack depths), the depth-to-width ratio a/c rises from 0.20 to 0.40 during the early propagation stage, leading to an 18–35% increase in the overall SIF (ΔK) at the crack front. The underlying mechanical mechanism can be attributed to deeper cracks (higher a/c) generating stronger three-dimensional constraint effects in the plate thickness direction [12], where the crack-tip stress field is dominated by normal principal stresses, driving a significant increase in ΔK_I_ (see Figure 18a).

For C1, C3, and C5 (with increasing initial crack widths), the depth-to-width ratio decreases from 0.375 to 0.25. This results in an approximate 12% rise in ΔK_I_ at the central crack front due to enhanced shallow shear stress components, while transverse stress relaxation slightly reduces ΔK_I_ at the crack ends (see Figure 18b). This phenomenon aligns with Irwin’s stress intensity factor distribution theory [8]: increasing the initial crack width flattens the stress gradient along the crack’s major axis, thereby mitigating stress concentration at the crack tips. Additionally, a reduced a/c weakens the crack closure effect [10], accelerating propagation rates in the central region.

In summary, variations in the initial crack size modulate the spatial distribution characteristics of the crack-tip stress field (principal stress orientation and gradient), thereby governing ΔK magnitudes. Among these parameters, the depth-to-width ratio (a/c) plays a dominant role in regulating crack driving forces.

**Figure 19:**
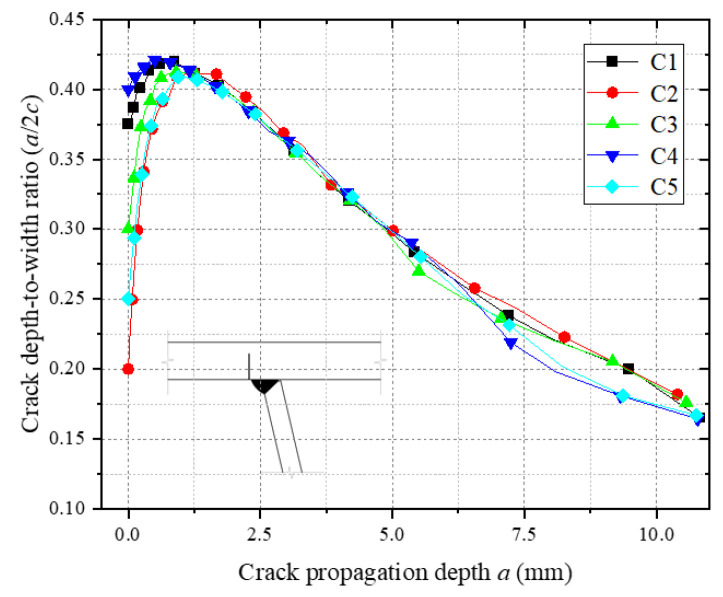
Depth-to-width ratio of crack propagation for different crack sizes.

Figure 19 illustrates the evolution of the depth-to-width ratios (a/c) during crack propagation for initial cracks of different sizes. As shown, all cracks exhibit similar trends in a/c variation: the ratio initially increases, reaches a peak level, and subsequently decreases. This indicates that fatigue cracks predominantly propagate along the depth direction during the initial stage. Once a critical depth is achieved, crack growth transitions to the width direction. The fundamentally identical a/c evolution trends across different initial crack sizes demonstrate that, under identical loading conditions and geometric constraints, cracks with varying initial dimensions converge toward similar propagation paths.

**Figure 20:**
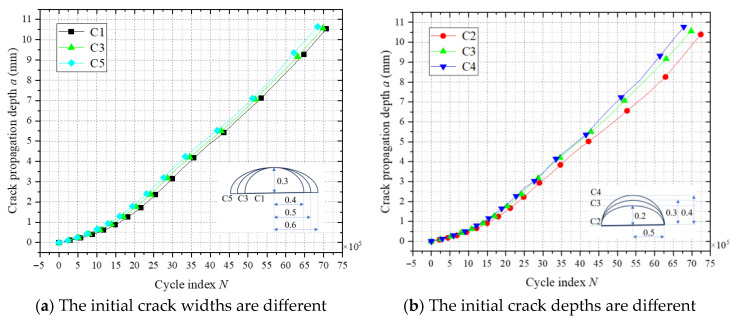
Crack propagation a-N for different initial crack sizes.

Figure 20 illustrates the relationship between the crack propagation depth and cycle count (a-N) for different initial crack sizes. As shown in Figure 20a, an increase in the initial crack width (C1, C3, C5) leads to a slight reduction in the crack propagation life, suggesting that fatigue life is only marginally sensitive to crack width. This phenomenon arises from the reduced depth-to-width ratio (a/c), which elevates ΔK at the central crack front due to enhanced shallow shear stress components while marginally lowering ΔK at the crack ends via transverse stress relaxation. The overall driving force for crack growth remains largely unchanged, resulting in negligible degradation of fatigue life. In contrast, Figure 20b demonstrates that increasing the initial crack depth (C2, C3, C4) significantly shortens the crack propagation life. The primary mechanism lies in the increased initial crack depth elevating the a/c, which intensifies the normal principal stress field at the crack front, thereby accelerating crack propagation.

### 4.2. Fatigue Crack Propagation Analysis at the Top Plate Weld Toe

**Figure 21:**
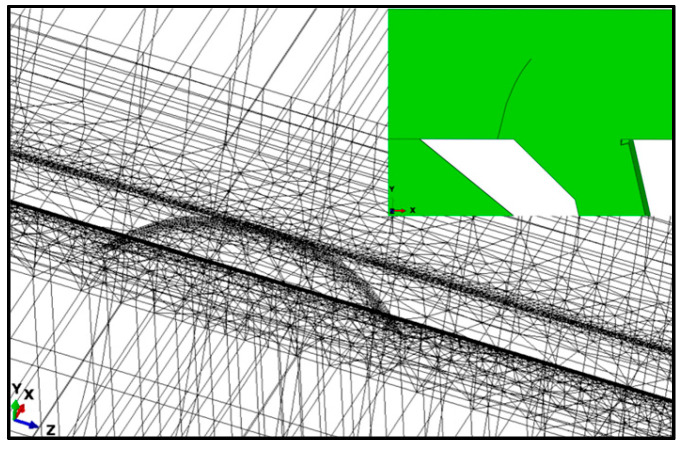
The crack propagation process of the top plate weld toe.

As illustrated in Figure 21, the fatigue crack propagation at the weld toe of the top plate was analyzed. With increasing load cycles, the crack primarily extends along the weld direction, while the crack width progressively increases. When the load cycles reach 6.98 million, the crack depth attains the critical dimension (two-thirds of the plate thickness), at which point the bridge deck is deemed to have undergone fatigue failure. During propagation, the crack exhibits not only thickness-direction growth but also a pronounced twisting phenomenon, gradually deviating toward the weld side. This behavior indicates that fatigue crack propagation is governed by multifactorial interactions, among which cyclic loading plays a pivotal role in crack evolution.

**Figure 22:**
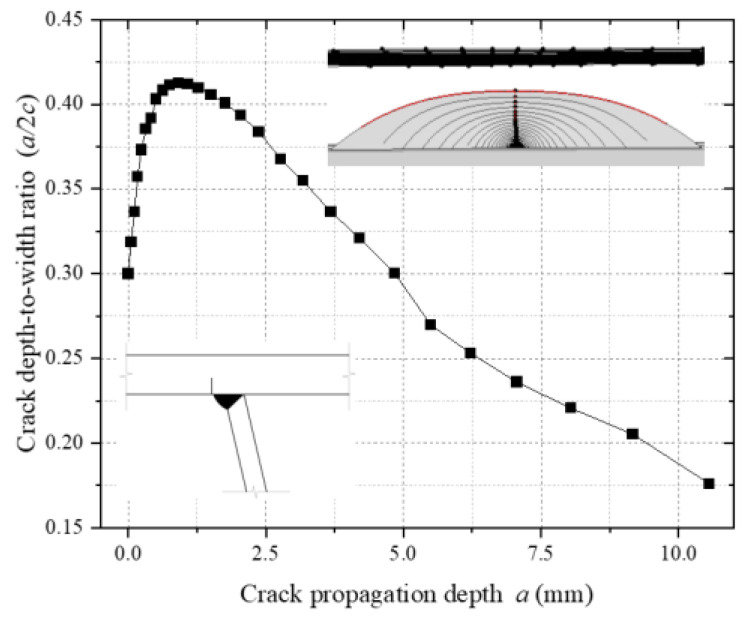
Depth-to-width ratio of weld toe cracks on the top plate.

Figure 22 illustrates the dynamic evolution of the crack depth-to-width ratio (a/c) at the top plate weld toe. Numerical analysis reveals that the initial a/c of 0.30 increases to 0.42 during the crack initiation phase. During this stage, crack propagation is dominated by growth in the thickness direction, driven by the combined effects of residual tensile stress and vehicular normal principal stress [12], which promote Mode I (tensile) crack opening. As propagation progresses into the mid-to-late stages, the a/c continuously decreases to below 0.20, indicating enhanced shear stress components (Mode II) that govern crack face sliding and redirect propagation toward the width direction. This staged variation in the a/c elucidates the transition mechanism of the crack-tip stress field from normal principal stress dominance to shear stress dominance.

**Figure 23:**
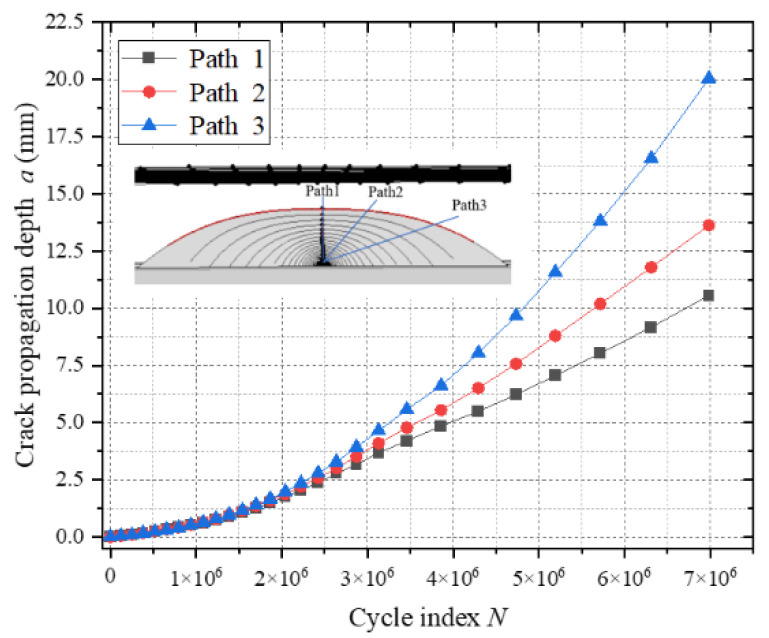
Fatigue crack propagation under different paths a-N.

Figure 23 presents a comparative analysis of the fatigue crack extension depth–cycle count (a-N) curves for cracks at the top plate weld toe. The analytical results demonstrate that path 1 exhibits the shortest crack extension length, while path 3 displays the longest, indicating significant differences in the crack propagation rates across distinct paths. However, the final fatigue lives of all paths converge to comparable values. Based on the principles of fracture mechanics parameter consistency, path 1—characterized by the fastest crack propagation rate—is selected as the reference path for fatigue life calculation.

In crack propagation analysis, K_I_, K_II_, and K_III_ denote the stress intensity factors corresponding to Mode I (opening), Mode II (sliding), and Mode III (tearing) crack propagation, respectively. Figure 16 illustrates the evolution of stress intensity factors during fatigue crack propagation at the top plate weld toe.

**Figure 24:**
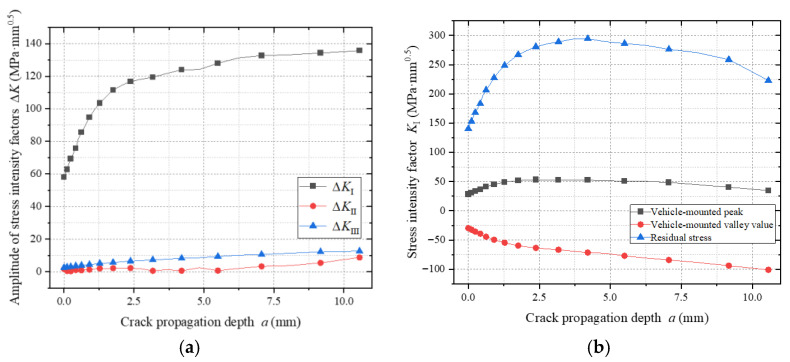
Stress intensity factor during the propagation of weld toe cracks in the top plate. (**a**) Amplitudes of stress intensity factors of different types. (**b**) Stress intensity factors under different loads.

As shown in Figure 24a, the stress intensity factor amplitude ΔK_I_ significantly exceeds ΔK_II_ and ΔK_III_, indicating that crack propagation is predominantly governed by Mode I (tensile opening). This model employs non-proportional cyclic loading to calculate ΔK by combining maximum and minimum stress states.

Figure 24b reveals that during peak vehicular loading (maximum stress condition), ΔK exhibits an initial increase followed by a decrease. This trend arises as the crack front extends from the tensile zone to the compressive zone: residual tensile stresses in the lower plate combine with external loads, amplifying ΔK; compressive stresses suppress crack-tip driving forces, reducing ΔK.

Under minimum vehicular loading, wheel loads act directly above the crack, forming localized compressive zones that continuously diminish ΔK. Residual stress distribution analysis demonstrates that the gradient field formed by residual tensile stresses in the lower plate and residual compressive stresses in the upper plate causes K-values associated with residual stresses to follow an initial rise and subsequent decline during propagation.

### 4.3. Impact of Residual Stresses on Fatigue Crack Propagation

To investigate the effects of residual stresses on fatigue crack propagation in weathering steel bridge deck welded joints, finite element simulations considering and disregarding residual stresses were performed for two critical locations: the top plate weld toe and longitudinal rib butt-joint vulnerable zone 1. The models were designated as DT2 (residual stress included), DF2 (residual stress excluded), CT2 (residual stress included), and CF2 (residual stress excluded). Additionally, to explore the influence of different loading conditions, simulations of crack propagation at the top plate weld toe under mid-span loading (DT2, DF2) and right-eccentric loading (DT3, DF3) were conducted. Details of these fatigue crack propagation models are summarized in Table 5.

**Figure 25:**
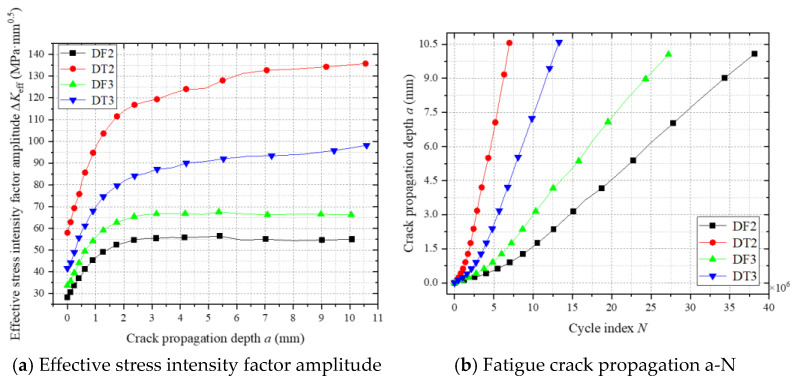
The fatigue crack propagation process at the weld toe of the roof panel.

Figure 25 reveals the relationship between the effective stress intensity factor amplitude (ΔK) and crack propagation a-N curves at the top plate weld toe. As shown in Figure 25a, ΔK does not exhibit unlimited growth, regardless of residual stress inclusion. During the initial propagation phase, ΔK increases rapidly due to the crack tip residing in the tensile zone. As the crack extends into the compressive zone, the growth rate decelerates.

Figure 25b demonstrates that fatigue lives under residual stress-included conditions (DT2, DT3) are reduced to 18.3% and 48.9% of those without residual stress (DF2, DF3). Mechanistic analysis reveals that residual tensile stresses (exceeding vehicular stresses) counteract compressive components in load cycles, elevating the mean stress level and amplifying the effective stress amplitude, thereby accelerating crack propagation. Additionally, mid-span loading exhibits a shorter fatigue life compared to right-eccentric loading due to its 34% higher cyclic stress amplitude, quantitatively validating the detrimental effects of residual stress and load position sensitivity.

These findings provide quantitative validation of residual stress effects on fatigue deterioration mechanisms, particularly critical for bridge decks where welding-induced residual stresses interact with complex vehicular load spectra. The ΔK evolution patterns offer direct guidance for optimizing weld sequence designs to generate beneficial compressive residual stresses in high-stress zones.

**Figure 26:**
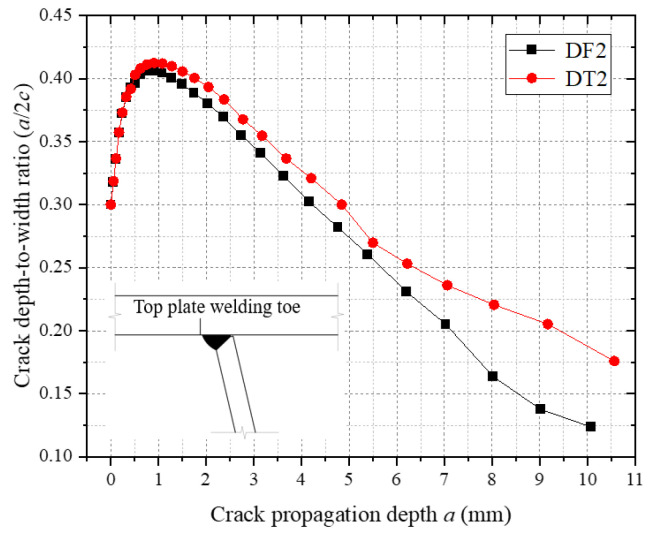
Depth-to-width ratio of the weld toe crack on the roof panel.

Figure 26 reveals the evolution of the crack depth-to-width ratio (a/c) at the top plate weld toe under residual stress-included and -excluded conditions. The a/c ratio exhibits a rising–peak–decay trend regardless of residual stress inclusion, indicating thickness-dominated propagation during the initial phase and a gradual transition to weld longitudinal extension in later stages. The comparative analysis shows that the a/c during intermediate and final propagation phases under residual stress conditions is 15–20% higher than in scenarios without residual stress. This confirms that residual stresses amplify normal stress components at the crack tip, sustaining higher depth-to-width ratios and altering spatial crack path distributions.

**Figure 27:**
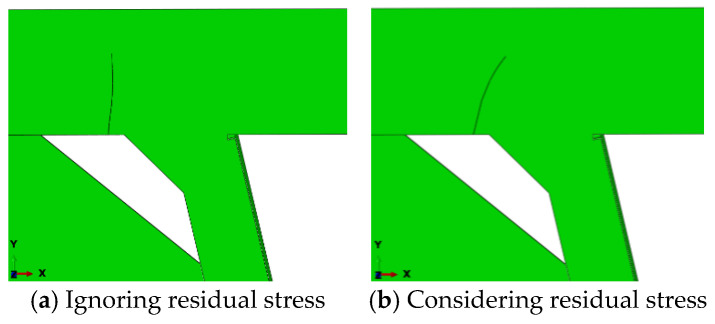
Fatigue crack morphology of the top plate weld toe.

Figure 27 contrasts fatigue crack morphologies with and without residual stress. Without residual stress, cracks propagate vertically upward, while residual stress induces 15–20° lateral deflection toward the weld toe. Numerical simulations attribute this directional shift to asymmetric stress fields generated by welding residual stresses, which redistribute crack-tip stress intensity factors (ΔK_I_, ΔK_II_) and reorient principal stress directions. Mechanistically, residual stresses act as an equivalent bending moment on the crack plane, driving persistent path deviation. The observed unidirectional deflection aligns with welding process symmetry in this model, though asymmetric welding or loading conditions could induce alternating deflection patterns in practice.

These findings systematically characterize the dual role of residual stresses: modulating crack depth-to-width evolution through normal stress amplification and governing crack path deflection via asymmetric stress field coupling. The results highlight the necessity of incorporating residual stress effects in fatigue-resistant design protocols for weathering steel bridge decks.

**Figure 28:**
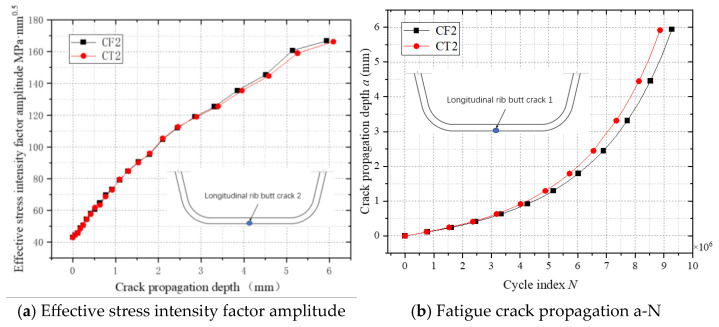
The fatigue crack propagation process of a vulnerable point in longitudinal rib butt joint.

Figure 28 illustrates the relationship between the effective stress intensity factor amplitude (ΔK) and crack propagation a-N curves in longitudinal rib butt-joint vulnerable zone 1. As shown in Figure 28a, the ΔK curves for scenarios with and without residual stress almost overlap, indicating minimal influence of residual stress on stress intensity factor amplitudes. Figure 28b reveals that the fatigue crack propagation life under residual stress conditions is reduced by approximately 6% compared to scenarios without residual stress. This limited impact stems from the uniaxial tensile stress cycles at the longitudinal rib butt joint, where residual stresses primarily alter the stress ratio at the crack front rather than amplifying ΔK. Unlike the top plate longitudinal rib joint subjected to tension–compression alternation, the longitudinal rib butt joint experiences predominantly tensile stress cycles, resulting in weaker coupling between residual stress and crack driving forces. The observed minor life reduction in the longitudinal rib butt joints quantitatively corroborates the localized interaction between residual stress fields and single-mode stress redistribution in butt-joint configurations.

### 4.4. Fatigue Crack Propagation at Other Vulnerable Locations

**Figure 29:**
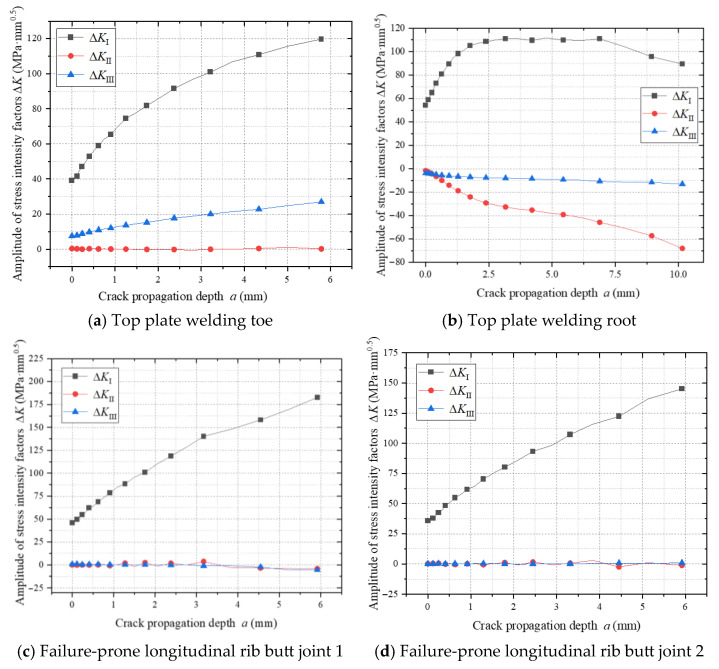
Amplitude of stress intensity factor during crack propagation at vulnerable locations.

Figure 29 illustrates the evolution of stress intensity factors (SIFs) at vulnerable welded joints in the weathering steel bridge deck. The top plate weld toe, longitudinal rib weld toe, and weld root regions exhibit Mode I-dominated mixed-mode propagation (ΔK_I_ > 85%, ΔK_II_ and ΔK_III_ ≤ 15%), where the crack-tip stress field is governed by the coupling of normal principal stresses and shear components (see Figure 29a,b). In contrast, longitudinal rib butt-joint vulnerable zones 1 and 2 (see Figure 17) display pure Mode I propagation (ΔK_II_, ΔK_III_ ≈ 0) due to negligible shear stress contributions (see Figure 29c,d).

The stress intensity factor amplitude ΔK_I_ at the top plate longitudinal rib joint exhibits a non-monotonic trend of rising–peak–decay (see Figure 29a). During the initial propagation phase, residual tensile stresses synergize with external tensile stresses, significantly amplifying ΔK_I_. As the crack extends into the compressive zone, compressive stresses weaken the crack driving force, decelerating ΔK_I_ growth in the intermediate phase. With further crack propagation, stress amplitude release in the flange gradually reduces ΔK_I_ during the final phase. This behavior reflects the dynamic interaction between residual stress fields, external loading, and crack-tip stress redistribution during fatigue crack propagation.

Conversely, ΔK_I_ in longitudinal rib butt-joint vulnerable zone 1 exhibits continuous growth, attributed to structural continuity suppressing compressive stress cycles and maintaining full-section tensile states in the flange, thereby enhancing stress accumulation at the crack tip. Residual stresses amplify the effective stress amplitude by 34% at top plate longitudinal rib joints through counteracting compressive stress components. However, at longitudinal rib butt joints subjected to uniaxial tensile cycles, residual stresses exert minimal regulatory effects on ΔK_I_.

**Figure 30:**
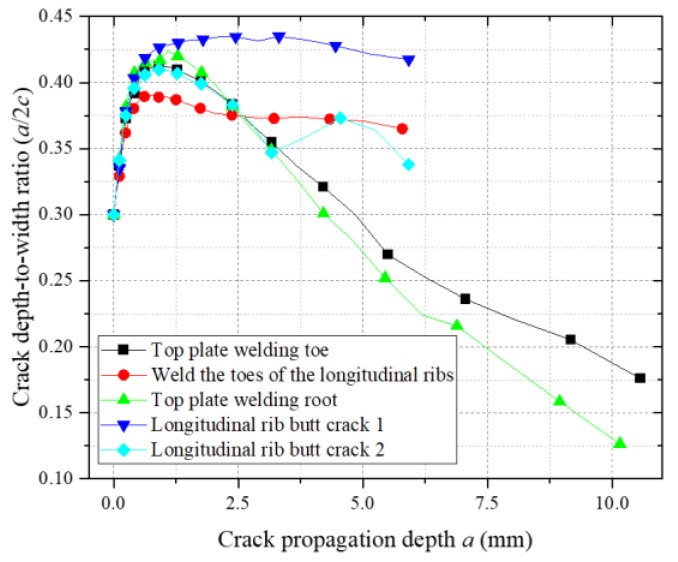
Depth-to-width ratio of crack propagation at vulnerable locations.

Figure 30 illustrates the depth-to-width ratio (a/c) evolution of fatigue cracks at vulnerable welded joints in the weathering steel bridge deck. All initial cracks exhibit a uniform depth-to-width ratio of 0.30. During the initial propagation phase, the a/c increases to 0.42, indicating thickness-dominated crack growth driven by the superposition of residual tensile stresses and externally applied normal principal stresses.

As the number of cycles increases, the a/c at the top plate weld toe, weld root, and longitudinal rib weld toe decreases below 0.20, reflecting a transition to shear stress-dominated propagation along the weld direction. Notably, longitudinal rib butt-joint vulnerable zone 1 maintains an a/c > 0.40 throughout propagation, confirming persistent principal stress control in the thickness direction. In contrast, longitudinal rib butt-joint vulnerable zone 2 exhibits an oscillatory a/c evolution (initial decrease followed by subsequent increase), attributed to its location at the flange–web geometric transition zone. Here, semi-elliptical crack morphology transitions to asymmetric branching, inducing multi-directional coupling effects in the crack-tip stress field and dynamic shifts in propagation modes.

**Figure 31:**
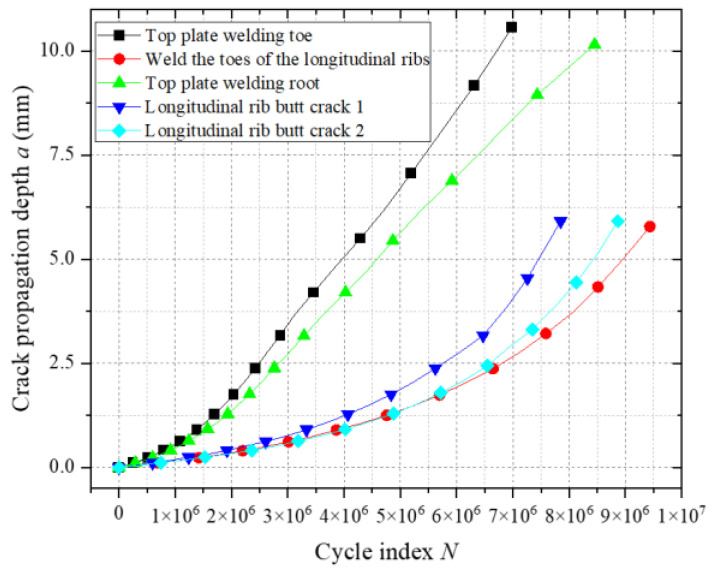
Fatigue crack propagation at vulnerable locations a-N.

**Figure 32:**
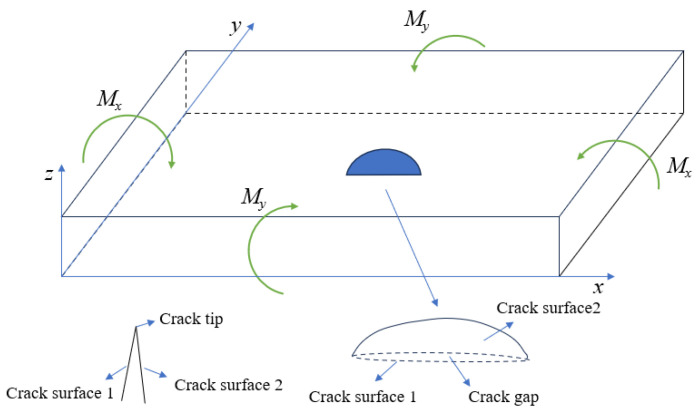
Illustration of the effect of bending moment on the crack propagation of the roof panel.

Figure 31 illustrates the fatigue crack propagation a-N relationships at vulnerable welded joints in the weathering steel bridge deck. Under mid-span loading, cracks at the top plate weld toe reach the critical crack size (two-thirds of the plate thickness) first, indicating that this location undergoes fatigue fracture earliest. Analysis of the crack growth rate (da/dN) curves reveals a monotonic increase in the growth rate with crack depth. Notably, the crack growth rates at the longitudinal rib weld toe and butt-joint vulnerable zones 1 and 2 are significantly higher than those at the top plate weld toe and weld root. This phenomenon arises from the biaxial bending effects in the bridge deck (see Figure 32). The top plate is subjected to biaxial bending moments (M_y_ and M_x_), where M_y_ (vertical bending) enhances the stress intensity factor amplitude at the crack tip, promoting propagation, while M_x_ (lateral bending) generates tensile stresses parallel to the crack plane, inducing crack closure effects that inhibit propagation. In contrast, longitudinal ribs experience uniaxial bending only, with no crack closure stresses, leading to higher growth rates. The coupling of biaxial bending reduces the crack growth rate at the top plate by approximately 35% compared to longitudinal ribs, consistent with the crack closure modulation mechanisms under biaxial stress reported in References [20,21,22].

## 5. Influence of Key Parameters on Fatigue Performance of Weathering Steel Bridge Decks

The comparative analysis of stress intensity factors at critical locations (top plate weld toe and longitudinal rib butt joints) confirms the distinct fatigue characteristics of the top plate weld toe, particularly in crack growth rate regularity and stress intensity factor distribution. Building on prior research [22], a single-variable parametric study was conducted using coupled Abaqus–Franc3D simulations under vehicular loading to isolate the effects of the initial crack size, top plate thickness, and wheel load pressure on fatigue life. The Paris law parameters for Q500ENH weathering steel at 20 °C were adopted, with C = 9.268 × 10^−11^ and m = 2.158, based on experimental calibration [23].

### 5.1. Top Plate Thickness Effects

**Figure 33:**
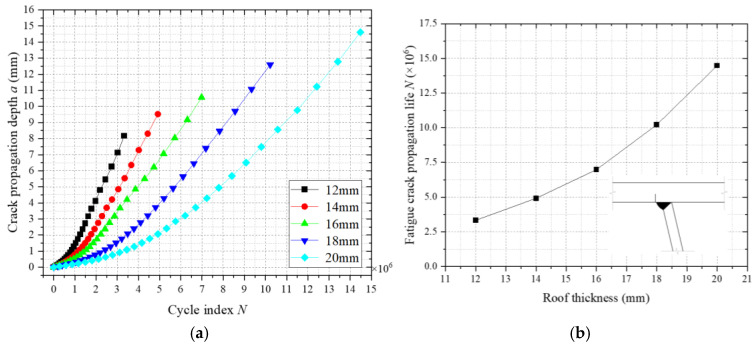
Influence of top plate thickness on fatigue crack propagation. (**a**) Fatigue crack propagation a-N. (**b**) The relationship between the thickness of the roof plate and the fatigue crack propagation life.

As a critical structural component, the top plate thickness significantly governs the fatigue resistance of weathering steel bridge decks. To investigate its influence on fatigue crack propagation at welded joints, numerical models with top plate thicknesses of 12 mm, 14 mm, 16 mm, 18 mm, and 20 mm were established (longitudinal rib thickness fixed at 8 mm). Figure 33a illustrates the fatigue crack propagation a-N relationships under varying thicknesses, while Figure 33b quantifies the correlation between thickness and fatigue life. The results demonstrate that increasing the thickness from 12 mm to 20 mm enhances the fatigue life by approximately 19% per 2 mm increment, with nonlinear amplification observed beyond 16 mm. The improvement arises from increased global stiffness, which reduces cyclic stress amplitudes and mitigates crack driving forces. A threshold thickness of 16 mm is identified, balancing structural efficiency and fatigue durability. The crack propagation simulations adopted critical crack depth criteria (two-thirds of plate thickness) and residual stress fields calibrated via thermal–mechanical coupling analyses. These findings provide actionable guidelines for optimizing fatigue performance in high-cycle-loading environments.

In the five weathering steel bridge deck models with varying top plate thicknesses, the critical crack size was defined as two-thirds of the plate thickness. As shown in Figure 33a, fatigue life increases significantly with greater top plate thickness, exhibiting an accelerating nonlinear trend. Within the studied thickness range (12–20 mm), each 2 mm increase in thickness enhances fatigue life by approximately 19%. This improvement arises from increased global stiffness due to thicker plates, which reduces nominal stress levels under identical cyclic loading, minimizes structural deflection, and diminishes cyclic stress amplitudes, collectively mitigating crack driving forces. Figure 33b indicates that fatigue life gains are marginal when the thickness is below 16 mm, suggesting a critical threshold for design optimization. Based on these findings, a minimum top plate thickness of 16 mm is recommended for weathering steel bridge decks with 8 mm thick longitudinal ribs, ensuring balanced structural efficiency and fatigue durability. The crack propagation simulations incorporated residual stress fields calibrated via thermomechanical coupling analyses, with adaptive meshing in Franc3D ensuring precise stress intensity factor (ΔK) calculations. The identified nonlinear relationship between thickness and fatigue life resolves uncertainties in fatigue design protocols for high-cycle-loading environments.

### 5.2. Impact of Wheel Load Pressure on Fatigue Crack Propagation

**Figure 34:**
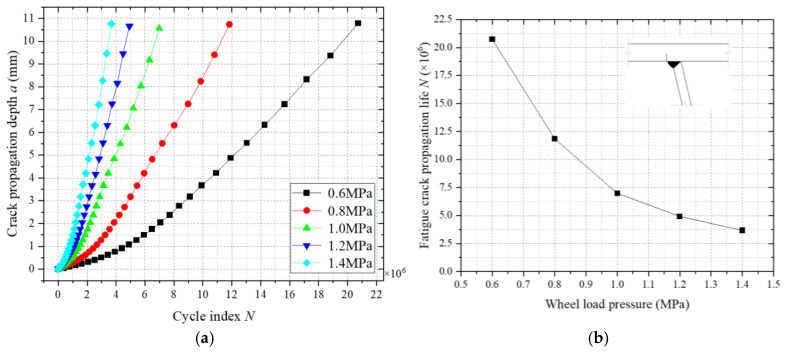
Influence of wheel load pressure on fatigue crack propagation. (**a**) Fatigue crack propagation a-N. (**b**) The relationship between wheel load pressure and fatigue crack propagation life.

To investigate the influence of wheel load pressure on fatigue crack propagation at welded joints of weathering steel bridge decks, numerical models with varying wheel load pressures (0.6 MPa, 0.8 MPa, 1.0 MPa, 1.2 MPa, and 1.4 MPa) were established under a constant load-bearing area. Figure 34a illustrates the fatigue crack propagation a-N relationships across these pressures, while Figure 34b quantifies the correlation between wheel load pressure and fatigue life. The results demonstrate that fatigue life decreases nonlinearly with increasing pressure, exhibiting a distinct stress threshold effect beyond 0.8 MPa, where the decay rate stabilizes. Specifically, the fatigue life at 1.4 MPa reduces to approximately 37% of that at 0.6 MPa, with the reduction rate decelerating progressively as the pressure rises. The nonlinear attenuation arises from intensified stress gradients at the crack front under higher pressures, amplifying stress intensity factor (ΔK) magnitudes while suppressing crack closure effects. These findings align with fracture mechanics principles, highlighting the critical role of stress amplitude modulation in fatigue design for heavy-load bridge decks.

## 6. Conclusions

This study proposes a coupled finite element simulation method for fatigue crack propagation at welded joints of weathering steel bridge decks, validated through experimental model calibration. By integrating Abaqus and Franc3D simulations, the fatigue crack growth process under the coupled effects of vehicular loading and residual stresses was systematically modeled, with the crack propagation life analyzed across five vulnerable locations. Comparative analyses of fatigue life under residual stress-included and -excluded conditions were conducted to quantify residual stress impacts. The key conclusions are as follows:

(1) At the top plate weld toe, longitudinal rib weld toe, and weld root, cracks exhibit Mode I-dominated mixed-mode propagation (ΔK_I_ > 85%, ΔK_II_ and ΔK_III_ ≤ 15%). The evolution of the depth-to-width ratio (a/c) reveals a transition mechanism in crack-tip stress fields from normal stress dominance to shear stress governance. In contrast, longitudinal rib butt-joint vulnerable zone 1 displays pure Mode I propagation (a/c > 0.40), with cracks consistently governed by through-thickness principal stresses. Vulnerable zone 2 exhibits a fluctuating a/c due to geometric discontinuity-induced stress coupling.
(2) Under mid-span loading, the crack growth rate at the top plate weld toe is 35% lower than that in longitudinal ribs under uniaxial loading. This results in 43.6% longer fatigue life, attributed to biaxial bending-induced crack closure effects (18% suppression by M_x_ components). The findings highlight the regulatory role of complex stress states in fatigue performance.
(3) For the deck-to-rib welded joints, residual stresses can elevate the effective stress intensity factor amplitude during crack propagation, thereby resulting in a reduction in the crack propagation life. However, in the case of rib splice welded joints, residual stresses exhibit a relatively minor influence on the effective stress intensity factor amplitude of crack propagation. Nevertheless, through the amplification of the stress ratio, residual stresses still contribute to a slight decrease in the fatigue life of the bridge deck, though the magnitude of this reduction remains limited. This differential behavior stems from distinct stress redistribution mechanisms and constraint conditions inherent to the two joint configurations under cyclic loading.
(4) In the study of the influence of different parameters on the fatigue crack propagation life of weathering steel bridge deck welded joints, it was found that the stress intensity factor of these joints increases with the growth in the initial crack dimensions. Specifically, increasing the depth and width of the initial cracks leads to a reduction in the crack propagation life, with crack depth exhibiting a more pronounced detrimental effect. Conversely, as the thickness of the top plate increases, the crack propagation life is extended, and the rate of this life extension accelerates progressively. Furthermore, an increase in the wheel load pressure elevates the stress intensity factor, thereby reducing the crack propagation life. Notably, the rate of this life reduction gradually decelerates with continued pressure amplification.

## Figures and Tables

**Table 1:** Chemical composition of Q500qENH weathering steel.

Chemical Component	C	Si	Mn	P	S	Cu	Cr	Ni	Nb	Mo	V	Al
Mass fraction (%)	0.07	0.35	1.24	0.010	0.001	0.31	0.51	0.31	0.027	0.05	0.040	0.027

**Table 2:** Welding heat source parameters for finite element welding simulation.

Welding Heat Source Parameters	U (V)	I (A)	V (mm/s)	η	a1	a2	b	c	$f_{f}/f_{r}$
Quantitative value	28	190	10	0.9	5	10	4	3	0.5

**Table 3:** Physical performance parameters of steel.

Temperature °C	Heat Conductivity Coefficient W/(m^2^·K)	Specific Heat J/kg·°C	Coefficient of Expansion (×10^−5^ m/°C)	Density kg/m^3^	Yield Stress MPa	Elasticity Modulus (×10^11^ Pa)
20	16.3	500	1.72	7800	235	2.05
100	17.5	500	1.75	7800	210	1.95
250	19.5	500	1.79	7800	175	1.87
500	21.5	500	1.84	7800	130	1.75
750	22.8	500	1.88	7800	40	1.6
1000	23.5	500	1.95	7800	25	1.5
1500	24.5	500	2.02	7800	2	1.25
2000	25	500	2.1	7800	0.5	1

**Table 4:** Initial crack configurations with different depth-to-width ratios.

ID	C1	C2	C3	C4	C5
Minor Axis Depth *a*_0_/mm	0.3	0.2	0.3	0.4	0.3
Major Axis Length *c*_0_/mm	0.4	0.5	0.5	0.5	0.6
Depth-to-Width Ratio	0.375	0.20	0.30	0.40	0.25

**Table 5:** Fatigue crack propagation models under different working conditions.

Model ID	Vulnerable Location	Loading Condition	Residual Stress Considered
DT2	Top Plate Weld Toe	Mid-Span Loading	Yes
DF2	Top Plate Weld Toe	Mid-Span Loading	No
DT3	Top Plate Weld Toe	Right-Eccentric Loading	Yes
DF3	Top Plate Weld Toe	Right-Eccentric Loading	No
CT2	Longitudinal Rib Butt-Joint Zone 1	Mid-Span Loading	Yes
CF2	Longitudinal Rib Butt-Joint Zone 1	Mid-Span Loading	No

## Data Availability

The original contributions presented in this study are included in the article. Further inquiries can be directed to the corresponding author.
